# Applicability of an Unmedicated Feeding Program Aimed to Reduce the Use of Antimicrobials in Nursery Piglets: Impact on Performance and Fecal Microbiota

**DOI:** 10.3390/ani10020242

**Published:** 2020-02-03

**Authors:** Paola López-Colom, Jordi Estellé, Jordi Bonet, Jaume Coma, Susana Ma. Martín-Orúe

**Affiliations:** 1 Animal Nutrition and Welfare Service, Animal and Food Science Department, Facultat de Veterinària, Universitat Autònoma de Barcelona, 08193 Bellaterra, Spain; paola.lopecol@gmail.com; 2 Facultad de Medicina Veterinaria y Zootecnia, Universidad Agraria del Ecuador, 090104 Guayaquil, Ecuador; 3 Génétique Animale et Biologie Intégrative (GABI), INRA, AgroParisTech, Université Paris-Saclay, 78350 Jouy-en-Josas, France; Jordi.Estelle@jouy.inra.fr or; 4 Vall Companys Group, 25191 Lleida, Spain; jbonet@vallcompanys.es (J.B.); jcoma@vallcompanys.es (J.C.)

**Keywords:** by-products, dietary fiber, fecal microbiota, gut health, in-feed antimicrobials, pig, post-weaning diarrhea, ZnO

## Abstract

**Simple Summary:**

The need for a reduction in the use of antibiotics in livestock to safeguard their efficacy requires the development of alternatives. In this line, the use of alternative by-products or ingredients, with functional properties brings the opportunity to improve pig health and thus, reduce medicalization. Therefore, in the present study, we aimed to evaluate the impact of an alternative feeding program based on unmedicalized diets formulated with fibrous by-products and functional feed ingredients on performance and fecal microbiota of young pigs compared to a common weaner diet supplemented with antibiotics. The alternative feeding program could anticipate the gut development of young piglets, which at the end of the nursery period presented a fecal microbiota more similar to that found in fattening animals. Moreover, piglets in the unmedicalized diets showed a trend to reduce the course of diarrhea immediately after weaning. The alternative feeding program showed, however, a reduced growth efficiency during the nursery period that needs to be discussed in the frame of the costs-benefits analysis of reducing antibiotics.

**Abstract:**

This study aimed to assess the impact of two different feeding programs, including or not antimicrobials, on gut microbiota development at early ages in commercial pigs. For this, 21-day-old weaned piglets were distributed into 12 pens (6 replicates with 26 pigs each) and fed ad libitum until fattening with: standard commercial formula with antibiotics and zinc oxide (2400 ppm) (AB), and alternative unmedicated feed formula (UN). Subsequently, the animals were moved to the fattening unit (F) receiving a common diet. Pigs were weighed, and feed consumption and diarrhea scores registered. Feces were collected on days 9 (pre-starter), 40 (starter) and 72 (fattening) post-weaning and microbial DNA extracted for 16S rDNA sequencing. Piglets fed UN diets had a worse feed efficiency (*p* < 0.05) than AB during nursery; however, UN pigs spent less time scouring after weaning (*p* = 0.098). The structure of fecal community evolved with the age of the animals (*p* = 0.001), and diet also showed to have a role, particularly in the starter period when UN microbiomes clustered apart from AB, resembling the ecosystems found in the fattening animals. Fibrolytic genera (*Fibrobacter*, *Butyrivibrio*, Christellansellaceae) were enriched in UN piglets whereas *Lactobacillus* characterized AB piglets (adjusted *p* < 0.05). Overall, this alternative feeding program could anticipate the gut development of piglets despite a lower feed efficiency compared to standard medicalized programs.

## 1. Introduction

Post-weaning diarrhea in piglets appears most frequently as a result of the intestinal dysbiosis associated with the multiple stressors of this stage that increase the opportunity for enteropathogens to thrive [1,2]. To prevent intestinal disorders, the use of medicalized diets with a prophylactic-metaphylactic purpose is a common practice in nurseries. However, the increased risk of developing antimicrobial resistances, associated with the widespread use of antibiotics, has led the EU to implement more restrictive policies for their use in livestock (Regulation EC 4/2019). Trying to respond to this new challenge, the scientific community and the industry are searching for alternative strategies capable of improving pig health and diminishing the need of prophylactic and therapeutic antibiotics. 

Within the most commonly studied alternatives are those that consider the in-feed use of additives such as organic acids, probiotics, prebiotics, or phytogenics. Many of these additives have been shown to promote beneficial effects on the gut ecosystem particularly at weaning, when the microbiota has to face the risk of dysbiosis [3]. Between the positive effects reported, we can find improvements in digestibility, intestinal immunity, and the promotion of beneficial bacteria at the expense of harmful [4,5,6,7]. 

Together with in-feed additives, dietary fiber (DF) has gained attention as a strategy to improve the gut health of weanlings [8,9,10,11]. In addition, the use of fibrous by-products has recently gained interest as a means of reducing production costs [12] and diminishing the environmental impact of the agroindustry [13]. A wide variety of different compositional DF and sources of non-starch polysaccharides (NSP), purified or not, have been tested, although mostly in fattening (and finishing) pigs [14,15,16]. Most of the beneficial effects of DF on gut health are thought to be mediated by changes promoted directly or indirectly in the gastrointestinal microbiota. Modulation of the gut ecosystem could be mediated by the supply of new growth substrates for particular microorganims but also indirectly by changes promoted in the physicochemical characteristics of the digesta or in their transit time [17].

The recent development of new generation sequencing (NGS) technology, with affordable associated costs, has allowed improvement in the knowledge of the role played by in-feed additives and DF on the intestinal microbiota of pigs. Different works have been published illustrating the changes induced by the use of organic acids [18,19], probiotics [20,21], or different types or levels of fiber [22], although studies are still scarce and results are not always consistent. Moreover, the definition of what should be considered a well-balanced and robust microbial ecosystem during the weaning period is still to be determined. In this context, improving current knowledge of how different diets can impact the development of the intestinal microbiota at early ages could be of high relevance [23].

Despite the growing interest of the industry in this topic, to our knowledge, no controlled study has been published testing the feasibility of alternative unmedicated nursery feeding programs addressed to improve the establishment of the intestinal microbial in young pigs raised under field conditions. In this work, we hypothesized that the inclusion of high-fiber by-products, combined with alternative functional ingredients, would be an effective strategy for the reduction of antibiotic use in young pigs by favoring their gut development through a distinct maturation of the gut microbiota. Therefore, we conducted a trial in a commercial farm comparing these two dietary regimes, assessing their impact on growth, diarrhea, and fecal microbiota during the nursery and early fattening phases by using NGS of the 16S RNA gene.

## 2. Materials and Methods

This work was approved by the Animal Ethics Committee of the Universitat Autònoma de Barcelona with the code CEEAH 1406.

### 2.1. Pig Husbandry and Diets

A total of 312 crossbred female piglets (Piétrain × (Landrace × Large White)) weaned at 21 days coming from a commercial farm of 2020 sows (Vall Companys S.A.) were moved to a nursery unit (Arbeca, Lleida, Spain) and distributed into 12 pens (26 piglets/pen) within the same shed. Distribution was made according to initial bodyweight (BW) at weaning (average of 5.78 ± 1.108 kg) conforming three different weight-blocks (heavy: 7.13 ± 0.022 kg; mid: 5.65 ± 0.031 kg; light: 4.55 ± 0.101 kg).

Two days after arriving at the nursery, all piglets were vaccinated for circovirus (Porcilis, Intervet). The nursery unit was equipped with central heating and forced ventilation with a cooling system and completely slatted plastic floors. Each pen was equipped with a nipple water drinker and a commercial hopper feeder. When clinical signs of diarrhea were observed, individual therapeutic treatments were provided by an IM injection (2 mL per 3 days) of enrofloxacin (Enrovall, Mevet, Lleida, Spain), benzylpenicillin plus dihydrostreptomycin (Glucilin-D, Laboratorios Iven, Madrid, Spain), and dexamethasone (Cordexvall, Mevet). Afterwards, animals were moved to an external fattening–finishing unit maintaining the nursery groups. This unit was equipped with natural ventilation and two-thirds slatted concrete floors. Each fattening pen was equipped with a nipple drinker to guarantee free access to water. 

Pens were allocated in two different nursery feeding programs (6 pens/program with 26 pigs/pen) consisting of commercial standard diets with commonly used antimicrobials (Zn and antibiotics) (AB), or non-medicated alternative diets, enriched with high-fiber by-products and alternative natural or functional ingredients (coconut oil, yeast, lactic acid) (UN). Both programs included three different diets (prestarter I (PI) from weaning to day 12 post-weaning, prestarter II (PII) from day 12 to day 19 post-weaning, and starter (S) from day 19 to day 42 post-weaning). Once in the fattening–finishing unit, all animals received the same diet for 56 days (F) without antimicrobials. All diets were in pelleted form and were designed to cover pig nutritional requirements [24]. The ingredients and chemical composition of the different diets are presented in Table 1. 

### 2.2. Growth Performance and Clinical Records

At weaning, BW was recorded by pen whereas at F phase BW was recorded individually. Feed intake was recorded by pen on the same days to calculate average daily gain (ADG), average daily feed intake (ADFI) and gain:feed ratio (GF) during the nursery period.

The incidence of diarrhea was measured per pen, at 2-day intervals from weaning to day 42 post-weaning, based on the following fecal score scale: 0 = normal solid feces; 1 = soft feces; 2 = some diarrheic feces; and 3 = generalized diarrhea. Frequency of individual treatments and mortality rate were also registered during the nursery period.

### 2.3. Fecal Sampling and Microbiota Analysis

On days 9 (PI), 40 (S) and 72 (F) post-weaning, a pool of fresh feces was sampled per pen and stored at −80 °C until microbial DNA extraction. The pool was made from 4 different fresh stools of firm consistency collected from the pen floor. DNA was extracted and purified using the commercial QIAamp DNA Stool Mini Kit (Qiagen, West Sussex, UK) following manufacturer’s instructions, and the DNA concentration and purity were checked using the NanoDrop 1000 Spectrophotometer (Thermo Fisher, Wilmington, DE, USA). The DNA was finally eluted in 200 μL of Qiagen buffer AE and stored at −80 °C until use.

The V3–V4 region of the 16S rRNA gene was sequenced using the MiSeq® Reagent Kit v2 (500 cycles; MiSeq from Illumina, San Diego, CA, USA). Sequence reads were processed on the QIIME v.1.9.1 pipeline [25] with default settings. The Phred quality filtering of already demultiplexed sequences was set at a quality score of Q20. Reads were clustered to operational taxonomic units (OTUs) at a 97% sequence similarity and picked by the subsampling open reference approach [26] at 10% of sequences subsampled. Representative sequences were assigned to a taxonomy against the bacterial 16S GreenGenes v.13.8 reference database [27] at a 90% confidence threshold, and sequence alignment was obtained through uclust. Chimeric sequences were removed with ChimeraSlayer [28], and singletons and OTUs with relative abundance across all samples below 0.005% were removed as recommended by Bokulich et al. [29]. The raw sequences are openly available in the European Nucleotide Archive (ENA) at https://www.ebi.ac.uk/ena, accession number PRJEB30501.

### 2.4. Statistical Analysis

The effect of the experimental treatments on growth performance was analyzed using R v3.4.3 and the stats package [30] for ANOVA including the diet as a fixed effect. The effect of the feeding programs on frequencies of individual treatments and mortality rates was also analyzed using the stats package and implementing the Fisher test. For all analyzed data, the pen was used as the experimental unit. The alpha level for the determination of significance for all the analyses was 0.05. The statistical trend was also considered for *p*-values > 0.05 and < 0.10 unless otherwise stated. Continuous data are presented as means and residual standard error (RSE).

For the biostatistical analysis of microbiota sequencing, the OTU table was imported to R with the phyloseq package [31]. Diversity was analyzed at OTU level using the vegan package (https://CRAN.R-project.org/package=vegan). Richness was calculated with raw counts and alpha diversity based on the Shannon index. To compare any differential effects from treatments, an ANOVA analysis was performed for richness and diversity with diet and age and their interaction considered as fixed effects. Multiple comparisons were performed under the Tukey adjustment method when any effect was observed (*p* < 0.05). In parallel, sequences were rarefied to visualize saturation of richness. Beta diversity analysis was conducted with calculation of the dissimilarity matrices based on Bray–Curtis distances and ordinated on a non-linear multidimensional scaling (NMDS) using the vegan package. An analysis of similarities (ANOSIM) was performed to compare dissimilarities between group and within groups (treatments and ages) and a permutational multivariate analysis of variance (PERMANOVA) was used to determine if the centroids differed among groups (diets, ages, and their interaction). In view of the NMDS visualization, data subsets were created to compare further differences between diets at each age and between ages. Finally, differential abundance analysis was performed with the taxa relative abundances because of the compositional nature [32] under a zero-altered negative binomial or negative binomial model with pscl [33] and mass [34] packages, respectively, and corrected by false discovery rate (FDR). Diet and age and their interaction were considered fixed effects. In addition, orthogonal contrasts were performed when diet effect was observed (*p* < 0.10) to compare differences between diets at PI, S and F ages, and thereafter adjusted under the Bonferroni method. Additionally, multiple comparisons between ages were performed under the Tukey adjustment method when an age effect was observed (*p* < 0.10). Both Bonferroni and Tukey adjustments were performed using the multcomp package [35].

## 3. Results

Globally, animals performed as expected and displayed a generally healthy status. Mortality was low, with three and five casualties during the nursery in groups AB and UN, respectively, with no differences between treatments (*p* = 0.723). Similarly, during the fattening period, two and one casualties were registered in AB and UN groups (*p* = 1.000), respectively. However, it needs to be noted that respiratory problems had to be treated over days 16 to 21 post-weaning by administering doxycycline in water (200 g Doxivall/L, Mevet, Lleida, Spain) to the entire set of animals included in the study.

### 3.1. Growth Performance and Clinical Response

The results of the growth performance of piglets along the nursery phase are presented in Table 2. Average feed intake was unaffected by the experimental treatments. Regarding growth, ADG was lower in UN-fed piglets compared to the AB group during PI. Additionally, GF was impaired in UN animals compared to AB ones over the PI period; however, UN animals showed improved GF during the PII compared to piglets supplemented with AB. Considering the whole nursery period, GF was shown to be worse in the UN-fed piglets than the AB ones.

Regarding incidence of diarrheas, only mild courses were observed during the PI period but none from day 12 post-weaning onwards. Therefore, the presence or absence of diarrhea (considered as scores of 1, 2, and 3) was evaluated as a percentage of days a pen presented diarrhea over a total of 12 days that scores were observed within the PI period (with an ANOVA). Regardless of the weaning BW (block), UN piglets tended to present diarrhea for a shorter time compared to AB animals (4.2% vs. 12.5% for UN and AB, respectively; RSE = 7.91, *p* = 0.098). At fattening, no differences were observed in diarrhea incidence (3.33% vs. 2.5% for UN and AB, respectively; RSE = 5.008, *p* = 0.714).

Frequencies of animals receiving individual therapeutic treatments (enrofloxacin, benzylpenicillin, and dexamethasone) are presented in Table 3. Fewer animals had to be treated over the entire nursery period within the mid-weight block (20 pigs) compared to heavy- (34 pigs) and light-weight blocks (51 pigs). Only the heavy-weight animals showed differences during the PI period related to treatments with more animals treated within the UN group (*p* = 0.046).

### 3.2. Fecal Microbiota 16S rRNA Gene Analysis

The average number of reads per sample differed among ages (*p* = 0.021), diverging PI (123,576 ± 71,572 no. reads/sample) and S (261,594 ± 66,268 no. reads/sample) ages (*p* = 0.017), showing fattening (185,621 ± 168,533 no. reads/sample) intermediate levels, whereas the number of reads for treatment was similar between treatments (202,678 ± 148,629 and 184,634 ± 94,450 no. reads/sample in AB and UN groups, respectively; *p* = 0.638). Nevertheless, all experimental groups similarly reached the plateau phase in rarefaction curves (Figure S1). 

Firmicutes, Bacteroidetes, Proteobacteria, and Spirochaetes were the major phyla (>1%), accounting on average, for 97.3% of the total groups detected. At lower ranks, Prevotellaceae, Ruminococcaceae, and Lachnospiraceae were the three most abundant families, accounting, on average, for almost half of the total abundance (48.9% of a total of 53 different families). In the case of genera, most of the sequences were not classified (46.9%), and *Prevotella* was the most prevalent genus (21.0%) among the total of 67 genera detected. The following most abundant genera were below 5%, including *Phascolarctobacterium* at 3.61% and *Lactobacillus* at 2.7%.

#### 3.2.1. Effects of Age and Diets on Microbial Ecosystem Structure and Diversity

**Figure 1:**
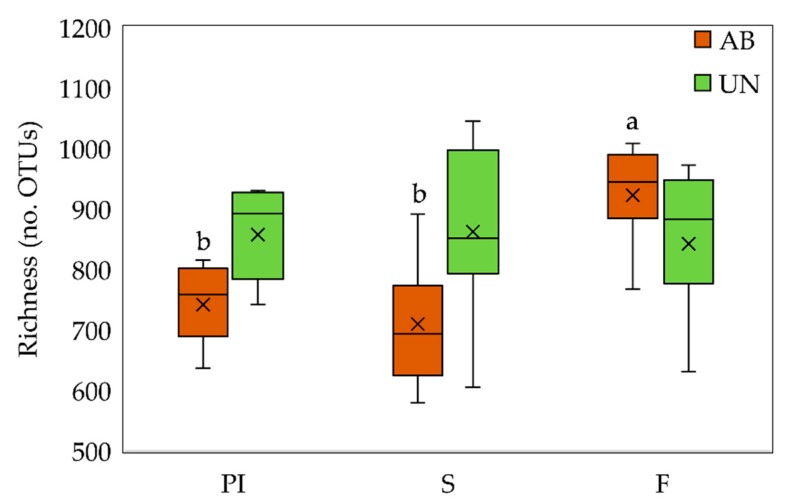
Richness (number of operational taxonomic units [OTU]) of fecal microbiota from animals fed with medicated diet (AB) and unmedicated diet (UN) during the nursery and fattening periods. In the fattening phase all animals received the same non-medicated diet. Sampling days included: prestarter I (PI) at 9 days post-weaning, starter (S) at 40 days post-weaning, and fattening (F) at 72 days post-weaning. ^a,b^ Different superscripts indicate statistical difference under Tukey adjustment within AB group (*p* < 0.05). No differences related to age were detected for the UN group.

A total of 903 different OTUs were shared among all groups, representing the 81.8% of all OTUs detected (Figure S2). No significant differences on richness were observed between diets at each time point (Figure 1); however, animals treated with AB presented lower richness during PI and S compared to the fattening phase (*p*_age_ = 0.003), whereas UN animals showed similar richness along the different phases (*p*_age_ = 0.472). Further differences were found between ages (*p* = 0.006) with fattening animals presenting a greater number of OTUs compared to PI and S animals (Tukey-adjusted *p* < 0.05).

**Figure 2:**
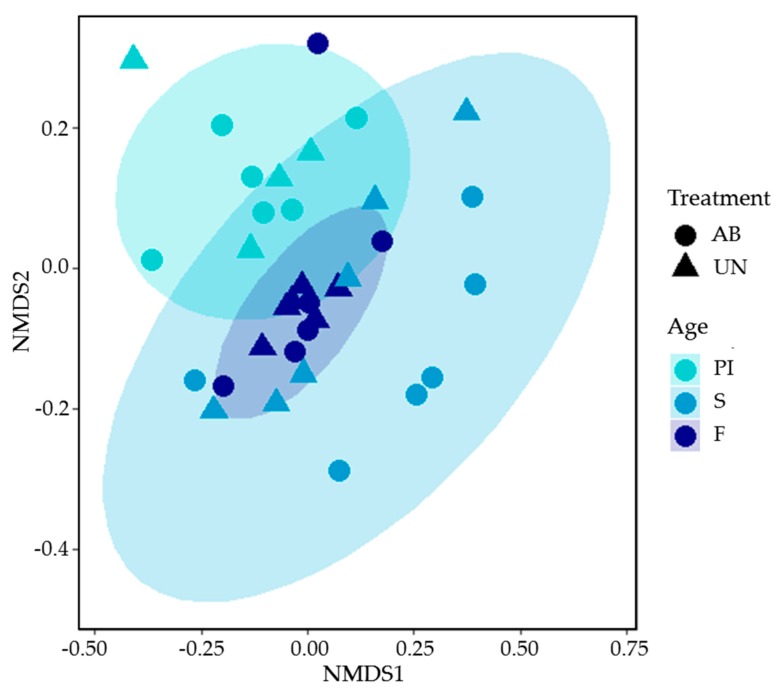
Non-metric multidimensional scaling (NMDS), based on the Bray–Curtis distance matrix and operational taxonomic unit (OTU) relative abundances of fecal microbiota from animals fed with medicated diet (AB) and unmedicated diet (UN) depending on age (prestarter I (PI) at 9 days post-weaning, starter (S) at 40 days post-weaning, and fattening (F) at 72 days post-weaning).

Regarding alpha diversity, no significant differences were detected between diets on any of the indices evaluated; however, alpha diversity was higher for the three indices in F compared to PI (Shannon: 5.20 ± 0.281 in F group and 4.70 ± 0.262 in PI group, *p* = 0.005; Simpson: 0.986 ± 0.005 in F group and 0.973 ± 0.009 in PI group, *p* = 0.024; and inverse Simpson: 78.5 ± 26.65 in F group and 41.4 ± 13.28 in PI group, *p* = 0.001) and S (Shannon: 5.20 ± 0.281 in F group and 4.75 ± 0.441 in S group, *p* = 0.008; Simpson: 0.986 ± 0.005 in F group and 0.975 ± 0.014 in S group, *p* = 0.042; and inverse Simpson: 78.5 ± 26.65 in F group and 50.6 ± 22.36 in S group, *p* = 0.011) time points. For the evaluation of the microbial ecosystem structure by NMDS ordination, results are displayed in Figure 2 and the supporting statistical analyses in Table A1 in Appendix A. ANOSIM analysis did not show statistical differences due to treatments when considering all phases (*p* = 0.167), although a slight trend (*p* = 0.108) was found at fattening age (Figure 2). In this regard, PERMANOVA analysis showed a trend for the interaction (*p* = 0.063) and Figure 2 shows how, during the S period, animals receiving the UN diet tended to cluster with those sampled at the F period, while those receiving the AB treatment clustered separately from PI and F animals. Microbiota ordination was also affected by age (*p* < 0.001), with S animals being differentiated from the rest (PERMANOVA *p* ≤ 0.001) and also PI from F (*p* < 0.035).

#### 3.2.2. Effects of Age and Diets on Differential Abundant Taxa

Regarding the effects of age and treatments on microbial groups, Table 4, Table 5 and Table 6 present the mean relative abundances of the most prevalent groups within each taxonomic rank including phylum, family, and genus, respectively. 

Most of the relevant changes on microbiota composition (considering adjusted-FDR *p*-values < 0.10) were observed with the age of the animals regardless of the treatment. At phylum level, Proteobacteria was observed to be reduced with age (Table 4). Regarding minor phyla, Verrumicrobia and also Cyanobacteria and TM7 were increased at older ages compared to PI, whereas Chlamydiae and Elusimicrobia were reduced (see Table S1 for minor populations).

At lower ranks (Table 5), the percentage of unclassified families was increased in S and F compared to the PI phase and Lactobacillaceae and Clostridiaceae also showed significant changes with age. Clostridiaceae were in higher proportions during F compared to younger ages, and genera within, such as *Clostridium* and SMB53 (Table S3), were shown also to be enriched with aging. Regarding minor families, increases with age were observed for Desulfovibrionaceae, Verrumicrobiaceae, Turicibacteraceae, and the corresponding genera, *Desulfovibrio*, *Akkermansia*, and *Turicibacter*, respectively, as well as F16 (for minor groups refer to Tables S2 and S3). Other minor genera (<1%), *Lachnospira*, YRC22, *Butyrivibrio*, and RFN20 were also increased at S and further. 

Other minor families (< 1%, Table S2) such as RFP12 and Coriobacteriaceae were also increased at F whereas Chlamydiaceae, accompanied by Chlamydia, Anaeroplasmataceae, Elusimicrobiaceae and Alcaligenaceae, and *Dorea*, *Collinsella* and *Helicobacter* were associated with PI animals and reduced thereafter. Interestingly, among them, Moraxellaceae (and *Acinetobacter* within) and Flavobacteriaceae reached abundances > 2% at PI but were subsequently decreased to values near 0%.

Regarding the effect of the treatments on particular microbial groups, most of the remarkable changes were observed at the PI phase. These include Bacteroidetes that were in lower proportion in UN-fed animals compared to AB during PI (Table 4). Nonetheless, the Firmicutes/Bacteroidetes (F/B) ratio was unaffected by the diet or age (*p* > 0.15). Within the Firmicutes phylum, Clostridiaceae were also maintained at lower percentages with the UN diet compared to AB. Regarding AB, Lactobacillaceae and *Lactobacillus* were higher during PI in AB than in UN piglets. On the contrary, Christensenellaceae was also remarkably higher in UN-fed pigs than in AB-fed ones, and especially at PI although it did not reach significance (*p* = 0.08).

When evaluating minor groups (<1%), Fibrobacteres and the corresponding family, Fibrobacteraceae, were higher in AB animals during the PI phase compared to UN-fed animals; however, these differences subsequently disappeared. Additionally, Streptococcaceae and *Streptococcus* were in higher proportions in AB-fed animals compared to UN-fed ones during the PI period. Similarly, Succinivibrionaceae and *Succinivibrio* were considerably higher with AB during the PI (*p* < 0.07) and also during the S period compared to UN diet. At S phase, Porphyromonadaceae were considerably higher with the AB compared to the UN diet.

On the other hand, with the UN diet, Cyanobacteria were in higher proportions compared to AB during the PI phase. Also, TM7 and F16 were in greater proportions in the UN group compared to the AB group at the PI phase. *Catenibacterium* was increased in UN animals during the PI and S periods, and *Butyrivibrio* was already increased with UN at the S phase compared to the AB diet, but not further. In addition, although UN animals harbored greater counts of Enterobacteriaceae at S, these were almost reduced at F compared to the AB diet (*p* = 0.065).

The rest of the changes were observed in very extreme minor groups (<0.10%). Nonetheless, it is worth mentioning that further differences, not covered by the statistical methods applied, could be contributing to the diverging microbial structures seen at S between treatments (see NMDS ordination in Figure 2). In this regard, except for a discordant sample within the UN group, in all UN-fed animals Chlamydiae, Elusimicrobia, Fibrobacteres, Planctomycetes, and WPS-2 were detected at S, whereas they were absent in AB animals.

## 4. Discussion

As stated above, the objective of this study was to assess, in nursery pigs, the potential of an unmedicalized feeding program formulated with fibrous by-products and functional ingredients, as an alternative to commercial programs that commonly include prophylactic antibiotics and pharmacological levels of zinc oxide (ZnO). In particular, we aimed to assess its effects under commercial field conditions, assuming those common practices and stressors that occur during this rearing period. From this point of view, the appearance during the PI phase of moderate courses of diarrhea, or during the PII phase of respiratory problems, could be considered as an intended part of the experimental design. Similarly, the required use of individual therapeutic treatments to control diarrhea episodes, or generalized treatment in water to treat respiratory problems, could be assumed as a frequent practice in commercial intensive farms. Despite this, the possible impact of these treatments in our results would deserve some discussion. Regarding the doxycycline treatment in water, its impact was expected to be similar in both experimental groups since it was performed in all the animals. Also, it is fair to point out that this treatment was administered between post-weaning days 16–21, almost three weeks before the fecal sampling within the S phase, therefore avoiding the short-term impact of the antibiotics. In the case of individual treatment by IM injection, it should be noted that most of the animals were treated in the PI period (anecdotally afterwards), and moreover, that the differences between groups did not reach statistical significance. Finally, it is important to remind that fecal samples collected from each pen were chosen from fresh stools with an average firm fecal consistency, being unlikely to include in these pools samples from treated animals. In summary, although we cannot discard effects of these treatments on the microbial ecosystem, the expected impact would not be of great magnitude, and eventually, it was similar in both dietary groups. 

Regarding the impact of experimental diets on performance of piglets, the UN diet was associated with a lower performance during the immediate post-weaning days (12 days; PI period) that animals seemed to recover in the subsequent PII period with higher feed intakes and improved GF. Although differences in the feed formula between treatments make difficult the discussion, we could try to find an explanation for the reduced growth during PI in the different nutrient composition of diets. In terms of energy supply, both diets were formulated above recommendations (2,448 kcal/kg) [24] and the greater amounts of net energy (NE) supplied by UN-PI diets would not explain the reduced growth but the lower ADFI registered for UN-treated animals in this period. Actually, when considering the feed intake and the energy content of each diet, the energy intake in terms of kcal/d was even higher for the UN diet than the AB diet (483 vs. 445 kcal/day for UN and AB, respectively). In terms of the protein content of diets, the trend was, however, opposite to the energy content with the PI-UN diets being formulated for a lower protein and in particular, a lower digestible lysine content than PI-AB diets. Actually, PI-UN diets showed digestible lysine levels below the recommendations (1.2–1.4%) [24] and when expressed relative to energy, differences were even higher between UN and AB diets (4.17 vs. 5.46 g digestible Lys/Mcal NE for UN and AB, respectively). This is the most plausible explanation for the impaired growth registered during the PI period with the UN diet. This lower protein content of the UN diet was somehow proposed as a safeguard in front of intestinal disorders. Using low protein diets during the post-weaning period is largely recognized as a strategy to control post-weaning diarrhea [36], although this could mean reduced growth of piglets during this period [37]. In any case, the impairment of performance registered in our trial during the PI with the UN diet could have been minimized by the compensatory growth registered during the subsequent PII period, and live weight differences were not eventually observed during the entire nursery period, or at fattening.

During the trial, the incidence of clinical diarrhea was low, making it difficult to assess the potential of these diets to reduce the incidence of post-weaning problems. Despite this, in the period immediately after weaning, a trend was observed in the UN treatment for shortened episodes of diarrhea compared to AB (*p* = 0.098). It is true that pigs from the heavy-weight block of the UN group received a higher number of therapeutic treatments than the AB group (Table 3), and this could confound the effects; however, it is fair to note that reductions in diarrhea episodes were also registered in the other weight blocks despite receiving similar antibiotic treatments. Together with a reduced protein level in the PI-UN diet, the functionality of other ingredients could also explain reduced scouring. Among them, coconut oil, yeast, or organic acids such as lactic acid, could have helped combat intestinal pathogens and ameliorated the vulnerability of young animals at weaning. Coconut oil represents a rapid energy source for younger and compromised animals [38,39] and it was only included in the UN diets (2–3%). In addition to being a rapid source of energy, coconut oil could also have provided antimicrobial activity against pathogens as previously reported *in vitro* [40]. In the same way, lactic acid has been demonstrated to be an effective organic acid to mitigate post-weaning diarrhea [41]. Yeast, besides being a protein and a vitamin source, can also act as a probiotic, improving fiber digestion and general gut health [21,42]. Moreover, the beta-glucans, which comprise its cell-wall, can act as prebiotics and have been shown to have immunomodulatory activities [43], which could also contribute to reducing post-weaning diarrhea and alleviating intestinal inflammation.

Associated with clinical and growth outcomes, fecal microbiota was evaluated as a main target for these alternative diets. Many authors have described the great impact of early-life events in mammals, and particularly in pigs, on their future health by shaping immune system development through changes in the pattern of microbial intestinal colonization [44,45]. Several works have demonstrated that aging involves greater richness and alpha diversity in the gut, and in the specific case of pig microbiota, the ecosystem has been described as becoming more homogenous between animals (reduced beta diversity) with age as of weaning [46,47,48]. These types of changes have also been observed in our study. The higher similitude of the microbiota structure in fattening animals could be related to the converging ecological successions that would have occurred in the different individuals. Additionally, the number of unknown groups, as reported by other authors [49], were shown to be increased with age.

Regarding changes in particular microbial groups, microbiota sequencing frequently looks for potential biomarkers that could be used as indices of gut health and maturity of the ecosystem. In this sense, the ideal sequence of consecutive niches during pigs’ early life is also stated as a target of research. In our study, regardless of the diet, microbial groups shifted according to the capability of animals to digest DF [46,48,50]. Common representative bacteria of rumen with fibrolytic activity were seen to increase with age, such as Fibrobacteres, *Butyrivibrio*, *Lachnospira*, YRC22, and *Megasphaera* [51,52]. *Fibrobacter* actually harbors the greatest cellulolytic activity [53]. Interestingly, some microbial groups, known for their capability to grow on intestinal mucus, also showed increase with age like the mucolytic bacteria *Akkermansia* and *Desulfovibrio* [54]. In contrast, Proteobacteria, despite numerically, appeared to be reduced with aging [47], together with other potential pathogens such as Chlamydiaceae, and *Helicobacter*.

Regarding the potential impact of the alternative UN diet on the pattern of intestinal colonization and microbiota structure, differential drifts were seen in specific groups during the nursery period. These changes can be globally seen through the NMDS visualization (Figure 2), whereby S pigs fed the UN diet and fatteners, under no experimental treatment, were clustered together. With these results, it could be hypothesized that a more mature fecal microbial community was promoted by the UN-starter diet, resembling that of fatteners. Actually, in terms of richness, while the AB diet animals showed an increase from S to F, the UN diet animals showed fairly constant richness values. This could be regarded as a beneficial effect of the UN treatment that would better prepare animals, and their intestinal microbiota, to face the dietary changes that they have to cope with when arriving at the fattening unit. Although it is difficult to attribute this effect to particular ingredients, considering the different feed formula used in this study, it is interesting to note the change of main cereals between treatments. While corn was the main cereal in the AB treatment (32–35%), in UN diets, corn was substituted by barley (30%). Barley is a whole cereal that is rich in insoluble fiber and soluble fiber compared to other cereals being particularly rich in beta-glucans. Beta-glucans from barley have been described as increasing viscosity of digesta and transit time in young pigs [15], which could have led to an increased amount of undigested material arriving at the hindgut with a more active fermentative activity. Barley has been described as favoring Firmicutes and Ruminococcaceae members such as *Dialister* or *Megasphaera* [15]; however, we could not find such changes in our study, probably due to the complexity of our diets and the interactions with other ingredients. It is worth to mention, additionally, that greatest divergences on gut microbiota ecosystems between corn- and barley-based diets have been detected at greater differences on cereal proportions and after longer times of administration than in our trial [55].

Together with the differences in main cereals, the introduction of wheat bran in the UN diets (4.5–2.5%) is remarkable. The inclusion of wheat by-products, such as wheat bran, rich in insoluble NSP, resistant to fermentation, can reduce bowel transit time [8], which could explain some of the changes found. Actually, wheat bran has been reported to reduce pathogen adhesion [56,57] and to promote the Prevotellaceae family [11,58]. Another non-energetic carbohydrate source that could have promoted changes in particular microbial groups is chicory pulp. This ingredient, introduced in PII-UN and S-UN diets, is rich in inulin, which is considered as a reference prebiotic [10]. Some of the microbial groups distinctive of UN-treated animals in the S and F periods are representatives of typical ruminal fibrolytic bacteria. This type of microorganisms requires complex and well-established ecosystems for their growth (like the rumen) as they rely on cross-feeding with other multiple microbial species. For instance, the increments of *Butyrivibrio* during S (also over ages), as an initial colonizer, might have facilitated the access of cellulose to other cellulolytic bacteria such as Fibrobacteres [53] and explained the increase we observed in the Fibrobacteriaceae family. Furthermore, it is interesting to see the considerably greater proportions of Christensenellaceae in UN animals, also found in rumen contents [52] and previously reported as being associated with a more competent digestion of carbohydrates in healthy and feed-efficient pigs [59]. Together with changes induced in microbial groups by the different ingredients provided by the experimental diets, different fermentable substrates could also have induced changes in the metabolic activity of the microbiota, unfortunately, we cannot give an answer to this question with our results.

Changes observed in microbial groups with diets could also be explained by the interference of antimicrobials on the normal microbiota development. During the first phase (PI), only ZnO was included in the AB diet and therefore changes reported in microbial groups could be related to this additive. In our study, at PI phase, Bacteroidetes and Fibrobacteres phyla were higher with the AB treatment than the UN one, but this effect disappeared at S and was opposite in the fattening period. Authors like Mukhopadhya et al. [43] also found a higher number of Bacteroidetes with high-doses of ZnO but not Fibrobacteres. Differences between studies could be explained by the different diets used to assess ZnO treatment. In our trial, PI diets from the two different feeding programs have a different formula, making it questionable whether to attribute the observed differences exclusively to ZnO. Regarding other changes promoted by AB treatment in microbial groups, the increase of *Lactobacillus* and its family observed from PI onwards is remarkable. This would apparently be contradictory to most published studies that show this group as being reduced by therapeutic doses of ZnO [9,60]. However, higher levels of *Lactobacillus* observed for the AB diet could be the result of an even greater relative decrease promoted by the UN in the lactobacilli population. A more fibrolytic ecosystem, prompted by a diet rich in fibrous ingredients, could have been associated with a smaller lactic acid population, such as that described in the rumen [61]. Moreover, it is also interesting to point out the lower proportions of Enterobacteriaceae seen in the S phase of the AB program coinciding with the introduction of antibiotics in the diets (amoxicillin, oxytetracycline, lincomycin), what suggests a direct impact of these antibiotics in these microbial taxa. However, all these differences found between feeding programs in structure, biodiversity or particular microbial taxa, did not persist or were minimized over the fattening phase when all animals clustered together. This would reinforce the resilience concept that has gained currency in the studies of microbiota and gut health [62]. This theory considers that external perturbations such as diet, or even the use of antimicrobials, might have limited impact on the gut microbiota once the ecosystem is mature and well-established.

## 5. Conclusions

The results from the present study indicate that using an alternative unmedicated feeding program during the nursery phase can promote an early maturation of intestinal microbiota compared to feeding programs including prophylactic antibiotics and pharmacological doses of ZnO. We hypothesized that this early maturation could favor the adaptation of animals to the fattening unit, with reduced episodes of diarrhea, although its cost would include a reduced growth efficiency during the nursery period. In addition, the results show that differences induced by these diets are transient, and that some weeks after, when the animals arrive at the fattening unit with the same diet, the ecosystems converge, confirming the high resilience of mature microbiota.

## Figures and Tables

**Table 1:** Ingredients and calculated chemical composition of the experimental diets ^1^ (as-fed basis).

	AB Diet			UN Diet			Common Diet
	PI	PII	S	PI	PII	S	F
Ingredient, %							
Corn	15.5	31.6	35.1	19.6		2.5	35.6
Barley	5.0	7.0			30.0	30.1	20.8
Wheat	14.5	16.5	27.6	2.0	17.7	22.0	
Wheat flour				10.0			
Rice	10.0			7.5			
Sorghum							7.7
Oat meal				10.0			
Whey powder sweet + Whey permeates + Yoghurt	18.0	10.0		14.2	5.6		
Glycerin + Sugar	2.3	2.5		5.0	4.0	3.0	
Bakery byproducts							3.5
Soybean meal + Extruded full soybean + Soybean concentrate	20.2	19.8	26.9	7.0	17.1	15.8	12.6
Sunflower meal							6.2
Fish meal	3.7	3.0					
Potato protein				2.0	2.0	3.0	
Wheat bran					4.5	2.5	
Wheat middling’s							5.6
Chicory pulp					2.5	2.5	
Soybean oil	2.5	2.0	2.8	3.1	3.1	2.2	1.8
Palm oil							2.0
Lard			1.3				0.3
Coconut oil				3.0	2.0	2.0	
Fish oil	0.4			0.5	0.5	0.5	
Plasma	3.6	1.4		3.0			
Mucosa hydrolysate 50%		1.5					
Yeast				5.0	5.0	5.0	
Formic + lactic acid			0.5				0.2
Lactic acid				0.5	0.5	0.5	
Premix ^2^	4.3	4.7	6.0	7.7	5.6	5.5	3.7
Antimicrobials, ppm							
ZnO	2400	2400	2400				
Amoxicillin			300				
Oxytetracycline			1000				
Lincomycin			1100				
Calculated chemical composition, %							
Dry matter	90.3	89.6	88.3	93.1	91.2	89.7	88.1
Crude protein	20.8	18.7	18.5	17.5	17.2	17.2	15.3
Crude fat	6.6	6.2	6.7	9.1	8.8	7.6	6.6
Crude fiber	2.3	2.7	3.0	2.5	5.1	5.1	4.4
d-Lys	1.4	1.3	1.2	1.2	1.1	1.1	1.0
NE pigs, kcal/kg	2555	2510	2492	2873	2582	2478	2463

^1^ Diets for medicated (AB) and unmedicated treatments (UN) over phases, which include prestarter I (PI) from weaning to 12 days post-weaning, prestarter II (PII) from day 12 to day 19 post-weaning, starter (S) from day 19 to day 42 post-weaning, and fattening (F) from day 42 to day 98 post-weaning (56 days at fattening unit). ^2^ Premix prestarter content: 10,000 IU vitamin A, 2000 IU vitamin D_3_, 75 mg vitamin E, 2 mg vitamin K, 2 mg vitamin B_1_, 4 mg vitamin B_2_, 6 mg vitamin B_6_, 0.03 mg vitamin B_12_, 25 mg vitamin C, 15 mg pantothenic acid, 25 mg nicotinic acid, 0.12 mg biotin, 1.0 mg folic acid, 180 mg betaine, 100 mg Fe, 160 mg Cu, 100 mg Zn, 50 mg Mn, 1.8 mg I, 0.3 mg Se; premix starter content: 10,000 IU vitamin A, 2000 IU vitamin D_3_, 30 mg vitamin E, 2 mg vitamin K, 2 mg vitamin B_1_, 4 mg vitamin B_2_, 3 mg vitamin B_6_, 0.03 mg vitamin B_12_, 20 mg vitamin C, 15 mg pantothenic acid, 25 mg nicotinic acid, 0.10 mg biotin, 0.5 mg folic acid, 100 mg Fe, 160 mg Cu, 100 mg Zn, 50 mg Mn, 1.8 mg I, 0.3 mg Se; and premix fattener content: 8000 IU vitamin A, 2000 IU vitamin D_3_, 20 mg vitamin E, 0.7 mg vitamin K, 1 mg vitamin B_1_, 4 mg vitamin B_2_, 1.2 mg vitamin B_6_, 0.02 mg vitamin B_12_, 12 mg pantothenic acid, 15 mg nicotinic acid, 100 mg Fe, 90 mg Cu, 100 mg Zn, 50 mg Mn, 1.8 mg I, 0.25 mg Se.

**Table 2:** Effect of experimental diets ^1^ on growth performance ^2^ of nursery piglets.

	Treatment			*p*-Value
	AB	UN	RSE	
PI period				
Final BW	7.82	7.44	1.232	0.603
ADG	170	138	0.010	<0.001
ADFI	174	168	0.014	0.478
GF	0.984	0.821	0.066	0.002
PII period				
Final BW	9.64	9.31	1.420	0.690
ADG	280	287	0.032	0.712
ADFI	378	364	0.049	0.626
GF	0.744	0.791	0.032	0.033
S period				
Final BW	20.1	19.4	2.184	0.581
ADG	447	430	0.034	0.424
ADFI	600	597	0.055	0.934
GF	0.745	0.721	0.020	0.059
Overall nursery				
ADG	341	322	0.026	0.239
ADFI	444	438	0.041	0.821
GF	0.770	0.736	0.020	0.014
Fattening period				
Final BW	52.9	51.6	7.60	0.139

^1^ Experimental treatments include medicated diet (AB) and unmedicated diet (UN) over phases that include prestarter I (PI) from weaning to 12 days post-weaning, prestarter II (PII) from day 12 to day 19 post-weaning, starter (S) from day 19 to day 42 post-weaning, and fattening (F) from day 42 to day 98 post-weaning (56 days at fattening unit). ^2^ Parameters include bodyweight (BW), average daily gain (ADG), average daily feed intake (ADFI), and gain:feed ratio (GF).

**Table 3:** Effect of experimental treatments ^1^ on number of nursery pigs receiving individual therapeutic treatments (enrofloxacin, benzylpenicillin, and dexamethasone). Values are presented for the different bodyweight (BW) blocks.

	Treatment		*p*-Value
	AB	UN	
PI period			
Heavy	9	19	0.046
Mid	8	7	1.000
Light	17	22	0.418
Total	34	48	0.094
PII period			
Heavy	2	1	1.000
Mid	2	1	1.000
Light	1	0	1.000
Total	5	2	0.448
S period			
Heavy	2	1	1.000
Mid	0	2	0.495
Light	5	6	1.000
Total	7	9	0.798
Overall	46	59	0.150

^1^ Experimental treatments include medicated diet (AB) and unmedicated diet (UN) over phases that include prestarter I (PI) from weaning to 12 days post-weaning, prestarter II (PII) from day 12 to day 19 post-weaning, starter (S) from day 19 to day 42 post-weaning, and fattening (F) from day 42 to day 98 post-weaning (56 days at fattening unit).

**Table 4:** Mean relative abundance (%) ^1^ of major phyla (> 1%) ^2^ by treatment ^3^ and age ^4^.

	PI		S		F		Adjusted *p*-Value ^5^		
	AB	UN	AB	UN	AB	UN	T	A	T × A
Firmicutes	48.5 ± 2.09	48.0 ± 6.20	49.4 ± 2.62	50.7 ± 2.52	52.6 ± 3.48	47.5 ± 1.22	0.620	0.790	0.537
Bacteroidetes	43.3 ± 1.42 ^a^	35.2 ± 1.74 ^b^	43.3 ± 2.95	40.4 ± 1.51	39.0 ± 1.96	41.8 ± 1.33	0.258	0.592	0.044
Spirochaetes	1.79 ± 0.602	1.80 ± 0.459	0.93 ± 0.456	3.10 ± 1.453	3.27 ± 0.968	5.52 ± 1.021	0.258	0.144	0.537
Proteobacteria	4.5 ± 3.34	12.7 ± 8.01	4.2 ± 1.87	2.5 ± 0.49	2.0 ± 0.39	2.2 ± 0.29	0.748	0.004	0.238

^1^ Standard error is presented in brackets. ^2^ Taxa are sorted by abundance. ^3^ Treatment includes medicated diet (AB) and unmedicalized diet (UN). ^4^ Age includes: prestarter I (PI) at day 9 post-weaning, starter (S) at day 40 post-weaning, and fattening (F) at day 72 post-weaning. ^5^ Effect of treatment (T), age (A), and treatment age interaction (T × A). ^a,b^ Superscripts indicate significant difference (*p* < 0.05) between diet AB and UN within age (PI, S, F). Performed by contrasts and corrected by Bonferroni adjustment.

**Table 5:** Mean relative abundance (%) ^1^ of major families (> 1%) ^2^ by treatment ^3^ and age ^4^.

	PI		S		F		Adjusted *p*-Value ^5^		
	AB	UN	AB	UN	AB	UN	T	A	T × A
Ruminococcaceae	20.5 ± 2.07	18.0 ± 3.69	20.0 ± 2.75	20.1 ± 2.22	19.9 ± 1.52	16.8 ± 0.60	0.471	0.676	0.780
Prevotellaceae	21.5 ± 2.13	15.2 ± 2.99	24.3 ± 4.97	23.6 ± 2.85	18.3 ± 2.04	20.5 ± 2.23	0.664	0.144	0.452
Unclassified	7.3 ± 1.02	9.2 ± 1.51 ^y^	14.9 ± 2.96	11.2 ± 1.15 ^x^	12.6 ± 1.09	11.7 ± 1.02 ^x^	0.652	0.001	0.257
Veillonellaceae	6.3 ± 1.17	7.1 ± 1.84	8.6 ± 1.82	11.0 ± 3.27	10.0 ± 0.91	9.1 ± 1.30	0.706	0.141	0.780
Lachnospiraceae	5.48 ± 0.404	5.49 ± 1.539	8.00 ± 1.845	8.40 ± 1.682	7.42 ± 1.392	7.12 ± 0.809	0.984	0.098	1.000
S24-7	12.3 ± 1.883	6.8 ± 2.19 ^x^	5.8 ± 0.96	6.5 ± 0.83 ^y^	6.6 ± 1.23	8.3 ± 0.63 ^y^	0.847	0.016	0.115
cand. Paraprevotellaceae	3.75 ± 0.591	3.41 ± 0.689	4.25 ± 0.803	4.09 ± 1.058	5.24 ± 1.104	4.97 ± 0.311	0.772	0.182	1.000
Lactobacillaceae	6.53 ± 1.569 ^a^	1.06 ± 0.595 ^b,x^	1.73 ± 0.76	0.69 ± 0.268 ^y^	3.40 ± 1.374	2.25 ± 0.608 ^x,y^	0.034	0.020	0.390
Spirochaetaceae	1.71 ± 0.583	1.68 ± 0.497	0.88 ± 0.433	3.00 ± 1.450	3.16 ± 0.958	5.43 ± 1.004	0.304	0.135	0.654
Erysipelotrichaceae	2.05 ± 0.682	1.97 ± 0.307	1.29 ± 0.236	1.77 ± 0.384	2.02 ± 0.493	1.48 ± 0.132	0.973	0.330	0.388
p-2534-18B5	0.87 ± 0.496	1.52 ± 0.643	0.29 ± 0.231	0.63 ± 0.228	2.00 ± 0.706	1.45 ± 0.385	0.731	0.143	0.803
Clostridiaceae	0.95 ± 0.153 ^a^	0.47 ± 0.038 ^by^	1.14 ± 0.153	1.12 ± 0.222 ^y^	1.84 ± 0.225	1.37 ± 0.179 ^x^	0.047	<0.001	0.152
Christensenellaceae	1.61 ± 0.566	7.96 ± 2.857	0.98 ± 0.570	2.92 ± 1.100	1.50 ± 0.271	3.47 ± 1.381	0.010	0.214	0.780

^1^ Standard error is presented in brackets. ^2^ Taxa are sorted by abundance. ^3^ Treatment includes medicated diet (AB) and unmedicated diet (UN). ^4^ Age includes phases from prestarter I (PI) at day 9 post-weaning, starter (S) at day 40 post-weaning, and fattening (F) at day 72 post-weaning. ^5^ Effect of treatment (T), age (A), and treatment age interaction (T × A). ^a,b^ Superscripts indicate significant difference (*p* < 0.05) between diet AB and UN within age (PI, S, F). Performed by contrasts and corrected by Bonferroni adjustment. ^x,y^ Superscripts indicate significant difference (*p* < 0.05) between ages (prestarter, starter, fattening) regardless of the diet. Corrected by Tukey adjustment.

**Table 6:** Mean relative abundance (%) ^1^ of major genera (>1%) ^2^ by treatment ^3^ and age ^4^.

	PI		S		F		Adjusted *p*-Value ^5^		
	AB	UN	AB	UN	AB	UN	T	A	T × A
Unclassified	44.7 ± 4.22	51.1 ± 4.45	42.8 ± 6.92	46.1 ± 4.46	49.3 ± 1.96	48.8 ± 2.46	0.716	0.587	0.914
*Prevotella*	21.6 ± 2.10	15.7 ± 2.90	24.4 ± 4.96	23.6 ± 2.85	18.3 ± 2.04	20.5 ± 2.23	0.739	0.178	0.573
*Phascolarctobacterium*	4.29 ± 0.377	4.18 ± 0.925	3.17 ± 0.704	2.76 ± 0.918	3.82 ± 0.411	3.62 ± 0.424	0.860	0.392	1.000
*Lactobacillus*	6.57 ± 1.566 ^a^	1.08 ± 0.609 ^b,x,y^	1.74 ± 0.761	0.69 ± 0.268 ^y^	3.40 ± 1.375	2.25 ± 0.608 ^x^	0.081	0.019	0.491
*Treponema*	1.71 ± 0.581	1.71 ± 0.476	0.89 ± 0.434	3.00 ± 1.451	3.16 ± 0.958	5.43 ± 1.004	0.331	0.147	0.753
*Megasphaera*	1.59 ± 1.003	1.94 ± 1.031	1.66 ± 0.994	3.76 ± 1.852	2.82 ± 0.727	2.04 ± 0.28	0.739	0.767	0.753
cand. *Prevotella*	1.45 ± 0.209	2.28 ± 0.537	2.39 ± 0.331	1.75 ± 0.574	2.06 ± 0.538	1.79 ± 0.151	0.989	0.822	0.466
CF231	2.05 ± 0.527	0.54 ± 0.177	1.08 ± 0.491	1.10 ± 0.344	1.74 ± 0.633	1.51 ± 0.178	0.331	0.438	0.242
*Oscillospira*	1.98 ± 0.335	1.44 ± 0.447 ^y^	3.85 ± 0.859	1.61 ± 0.344 ^x^	1.60 ± 0.045	1.75 ± 0.1 ^y^	0.167	0.042	0.140
*Ruminococcus*	1.19 ± 0.286	1.04 ± 0.229	1.15 ± 0.175	1.35 ± 0.305	1.31 ± 0.084	1.20 ± 0.137	0.989	0.841	0.878
*Roseburia*	0.85 ± 0.158	2.08 ± 0.828	3.19 ± 1.355	1.27 ± 0.3	1.26 ± 0.647	1.34 ± 0.58	0.989	0.260	0.149

^1^ Standard error is presented in brackets. ^2^ Taxa are sorted by abundance. ^3^ Treatment includes medicated diet (AB) and unmedicated diet (UN). ^4^ Age includes phases from prestarter I (PI) at day 9 post-weaning, starter (S) at day 40 post-weaning, and fattening (F) at day 72 post-weaning. ^5^ Effect of treatment (T), age (A), and treatment age interaction (T × A). ^a,b^ Superscripts indicate significant difference (*p* < 0.05) between diet AB and UN within age (PI, S, F). Performed by contrasts and corrected by Bonferroni adjustment. ^x,y^ Superscripts indicate significant difference (*p* < 0.05) between ages (prestarter, starter, fattening) regardless of the diet. Corrected by Tukey adjustment.

## Supplementary Materials

The following are available online at https://www.mdpi.com/2076-2615/10/2/242/s1: Figure S1: Rarefaction curves of animals fed with antimicrobial-supplemented diet (AB) and unmedicated diet (UN) depending on age (prestarter I [PI] at 9 days post-weaning, starter [S] at 40 days post-weaning, and fattening [F] at 72 days post-weaning); Figure S2: Venn diagram of total 1104 OTUs detected on animals fed with antimicrobial-supplemented diet (AB) and unmedicated diet (UN) depending on age (prestarter I [PI] at 9 days post-weaning, starter [S] at 40 days post-weaning, and fattening [F] at 72 days post-weaning); Table S1: Differential abundance of minor phyla (<1.0%) between treatments, ages, and their interaction; Table S2: Differential abundance of minor families (<1.0%) between treatments, ages, and their interaction; Table S3: Differential abundance of minor genera (<1.0%) between treatments, ages, and their interaction.

## Appendix A

**Table A1:** Analysis of similarities (ANOSIM) and permutational multivariate analysis of variance (PERMANOVA), based on Bray–Curtis distance matrices, to test experimental treatment ^1^ effect on microbial structure and homogeneity.

	ANOSIM ^2^		PERMANOVA ^3^	
	Statistic	Bonferroni *p*-Value	Statistic	Bonferroni *p*-Value
Treatment ^4^	0.031	0.167	1.811	0.025
UN vs AB at PI	0.143	0.522	1.397	0.293
UN vs AB at S	0.263	0.156	1.705	0.234
UN vs AB at F	0.124	0.108	1.358	0.246
Age ^5^	0.279	0.001	3.343	<0.001
S vs PI	0.323	0.006	3.136	<0.001
S vs F	0.162	0.015	2.312	0.001
F vs PI	0.470	0.003	4.476	0.035
Interaction	-	-	1.404	0.063

^1^ Experimental treatments include medicated diet (AB) and unmedicated diet (UN) during the nursery and fattening periods. In the fattening phase, all animals received the same non-medicalized diet. Sampling days included: prestarter I (PI) at 9 days post-weaning, starter (S) at 40 days post-weaning, and fattening (F) at 72 days post-weaning. ^2^ Treatment and age effects were obtained from separate independent ANOSIM tests. ^3^ Treatment, age, and interaction effects were obtained from a unique common two-way PERMANOVA test with interaction. ^4^ Further differences between treatments were performed with age subsets and *p*-values were corrected by Bonferroni adjustment. ^5^ Further differences between ages were performed with age subsets, irrespective of treatments, and *p*-values were corrected by Bonferroni adjustment.
